# A Hamilton–Jacobi-based proximal operator

**DOI:** 10.1073/pnas.2220469120

**Published:** 2023-03-29

**Authors:** Stanley Osher, Howard Heaton, Samy Wu Fung

**Affiliations:** ^a^Department of Mathematics, University of California, Los Angeles, CA 90095; ^b^Typal Research, Typal LLC, Los Angeles, CA 95811; ^c^Department of Applied Mathematics and Statistics, Colorado School of Mines, Golden, CO 80401

**Keywords:** proximal, Hamilton–Jacobi, Cole–Hopf, Moreau, zeroth-order

## Abstract

First-order optimization algorithms are widely used today. Two standard building blocks in these algorithms are proximal operators (proximals) and gradients. Although gradients can be computed for a wide array of functions, explicit proximal formulas are known for only limited classes of functions. We provide an algorithm, HJ-Prox, for accurately approximating such proximals. This is derived from a collection of relations between proximals, Moreau envelopes, Hamilton–Jacobi (HJ) equations, heat equations, and Monte Carlo sampling. In particular, HJ-Prox smoothly approximates the Moreau envelope and its gradient. The smoothness can be adjusted to act as a denoiser. Our approach applies even when functions are accessible only by (possibly noisy) black box samples. We show that HJ-Prox is effective numerically via several examples.

## Significance

Many objective functions do not admit explicit formulas for their proximal operators. Moreover, these operators often cannot be estimated using exact gradients (e.g., when objectives are accessible via an oracle). In this work, we give a formula for accurately approximating proximal operators using only (possibly noisy) objective function samples.

The rise of computational power and availability of big data brought great interest to first-order optimization methods. Second-order methods (e.g., Newton’s method) are effective with moderately sized problems but generally do not scale well due to memory requirements increasing quadratically with problem size and computation costs increasing cubically. First-order methods often comprise gradient and proximal operations, which are typically cheap to evaluate relative to problem size. Although gradients can be computed for many functions (or numerically approximated), the computation of proximals involves solving a small optimization problem. In special cases (e.g., with ℓ_1_ norms), these subproblems admit closed-form solutions that can be quickly evaluated (e.g., ref. 1). These formulas yield great utility in many applications. However, we are presently interested in the class of problems with (potentially nondifferentiable) objectives for which proximal formulas are unavailable.

We propose an approach to compute proximal operators and corresponding Moreau envelopes for functions *f*. We leverage the fact that the Moreau envelope of *f* is the solution to a Hamilton–Jacobi (HJ) equation (2). The core idea is to add artificial viscosity to HJ equations and obtain explicit formulas for the proximal and Moreau envelopes using Cole–Hopf transformation (2, section 4.5.2). This enables proximals and Moreau envelopes of smooth *f* to be approximated.

Our proposed proximal approximations (called HJ-Prox) are computed using only function evaluations and can, thus, be used in a zeroth-order fashion when integrated within an optimization algorithm. Finally, Monte Carlo sampling is employed to mitigate the curse of dimensionality when using HJ-Prox in dimensions higher than three. Numerical experiments show that HJ-Prox is effective when employed within optimization algorithms when the proximal is unavailable, including with black box oracles. Our work can generally be applied to first-order proximal-based algorithms such as the alternating direction method of multipliers (ADMM) and its variants (3–6) and operator splitting algorithms (7–11).

## Proximal Operators and Moreau Envelopes

Consider a function *f*: ℝ^*n*^ → ℝ and time *t* > 0. The proximal prox_*tf*_ and the Moreau envelope *u* of *f* (12, 13) are defined by

[1] $$\begin{array}{c} {\text{prox}}_{tf}(x)≜\underset{z\in {\mathbb{R}}^{n}}{\text{argmin}}\left\{f(z)+\frac{1}{2t}\|z-x{\|}^{2}\right\}, \end{array}$$

and

**Fig. 1.**
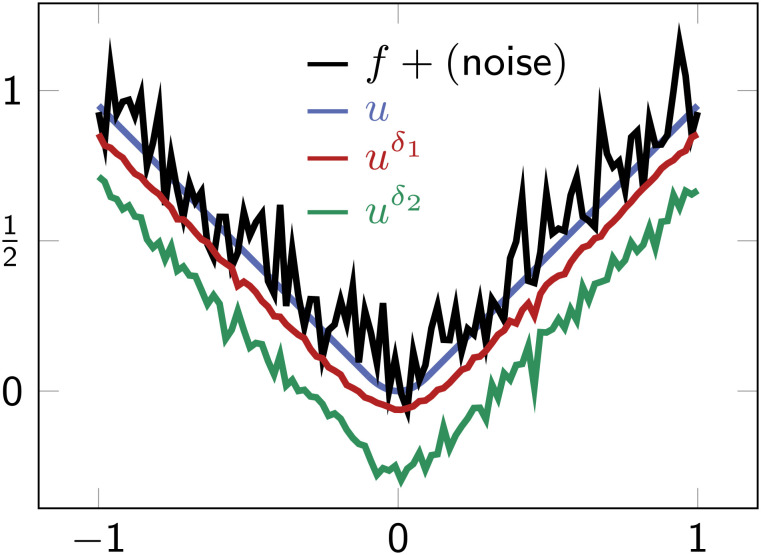
Moreau envelope approximation *u*^*δ*^ using only noisy function samples with *δ*_1_ = 0.1 and *δ*_2_ = 0.01. Here, *f*(*x*)=|*x*|+*ϵ*, with additive noise *ϵ* ∼ 𝒩(0, 0.1) in each function evaluation. To aid visualization, we include the envelope *u* (black line) of |*x*|. Here, larger *δ*_1_ better smooths noise and approximates the envelope *u*.

[2] $$u(x,t)≜\underset{z\in {\mathbb{R}}^{n}}{\min}\left\{f(z)+\frac{1}{2t}\|z-x{\|}^{2}\right\},$$

where ∥ ⋅ ∥ denotes the ℓ_2_ norm. The proximal is the set of minimizers defining the envelope. As shown in Fig. 1, the envelope *u* widens valleys of *f* while sharing global minimizers. A well-known result (e.g., refs. 1 and 14) states that if the envelope *u* is differentiable at *x*, then

[3] $$\begin{array}{c} \nabla u\left(x,t\right)=\frac{x-{\mathrm{prox}}_{\mathrm{tf}}\left(x\right)}{t}. \end{array}$$

Rearranging reveals

[4] $$\begin{array}{c} {\mathrm{prox}}_{\mathrm{tf}}\left(x\right)=x-t\nabla u\left(x,t\right). \end{array}$$

A key idea we use is to estimate the proximal for continuous *f* by replacing *u* with a smooth approximation *u*^*δ*^ ∈ *C*^∞^(ℝ^*n*^), derived from a Hamilton–Jacobi (HJ) equation.

## Hamilton–Jacobi Connection

The envelope *u* is a special case of the Hopf–Lax formula (2). Fix any time *T* > 0. For all times *t* ∈ [0, *T*], the envelope *u* is a viscosity solution e.g., (2, chapter 3, theorem 6) to the HJ equation

[5] $$\begin{array}{c} \left\{\begin{array}{ccc} u_{t}+\frac{1}{2}{\left\|\nabla u\right\|}^{2} & =0 & \;\text{in}\;R^{n}\times \left(0,T\right]\\ u & =f & \;\text{on}\;R^{n}\times \left\{t=0\right\}. \end{array}\right. \end{array}$$

Fixing *δ* > 0, the associated viscous HJ equation is

[6] $$\begin{array}{c} \left\{\begin{array}{ccc} u_{t}^{\delta }+\frac{1}{2}{\left\|\nabla u^{\delta }\right\|}^{2} & =\frac{\delta }{2}\Delta u^{\delta } & \;\text{in}\;R^{n}\times \left(0,T\right]\\ u^{\delta } & =f & \;\text{on}\;R^{n}\times \left\{t=0\right\}, \end{array}\right. \end{array}$$

where *Δu* is the Laplacian of *u*. If *f* is bounded and Lipschitz, Crandall and Lions (15) show *u*^*δ*^ approximates *u*, i.e., *u*^*δ*^ → *u* uniformly as *δ* → 0^+^.

## Cole–Hopf Transformation

Using the transformation *v*^*δ*^ ≜ exp(−*u*^*δ*^/*δ*), originally attributed to Cole and Hopf (2, 16), the function *v*^*δ*^ solves the heat equation, i.e.,

[7] $$\begin{array}{c} \left\{\begin{array}{ccc} v_{t}^{\delta }-\frac{\delta }{2}\Delta v^{\delta } & =0 & \;\text{in}\;R^{n}\times \left(0,T\right]\\ v^{\delta } & =\exp(-f/\delta ) & \;\text{on}\;R^{n}\times \left\{t=0\right\}. \end{array}\right. \end{array}$$

This transformation is of interest since *v*^*δ*^ can be expressed via the convolution formula (e.g., ref. 2 for a derivation)

[8a] $$\begin{array}{cc} v^{\delta }\left(x,t\right) & =(\Phi_{\delta t}*\exp\left(-f/\delta \right))\left(x\right) \end{array}$$

[8b] $$\begin{array}{cc} & =\int_{R^{n}}\Phi_{\delta t}\left(x-y\right)\exp\left(-f(y)/\delta \right)\;\text{d}y, \end{array}$$

where *Φ*_*δt*_ is a fundamental solution to Eq. **7**, i.e.,

[9] $$\begin{array}{c} {\text{Φ}}_{\delta t}(x)≜\left\{\begin{array}{ll} {\left(2\pi \delta t\right)}^{-n/2}\exp\left(-|x{|}^{2}/(2\delta t)\right) & \text{in}{\mathbb{R}}^{n}\times (0,\infty )\\ 0 & \text{otherwise.} \end{array}\right. \end{array}$$

Using algebraic manipulations, we recover the viscous solution

[10] $$\begin{array}{c} u^{\delta }\left(x,t\right)=-\delta \ln(\Phi_{\delta t}*\exp\left(-f/\delta \right))\left(x\right)\;\;\;\text{in}\;R^{n}\times \left(0,T\right]. \end{array}$$

Differentiating reveals

[11] $$\begin{array}{c} \nabla u^{\delta }\left(x,t\right)=-\delta \cdot \nabla \left[\ln\left(v^{\delta }\left(x,t\right)\right)\right]=-\delta \cdot \frac{\nabla v^{\delta }\left(x,t\right)}{v^{\delta }\left(x,t\right)}. \end{array}$$

## Monte Carlo Sampling

There are different ways to estimate the integral formula for *v*^*δ*^ in Eq. 8. For example, one may use a grid for numerical estimation, use uniform sampling, or potentially sample from exp(−*f*(*y*)/*δ*). However, we find that the most efficient way to estimate this *v*^*δ*^ is by writing this as an expectation with respect to a Gaussian, i.e.,

[12a] $$\begin{array}{cc} v^{\delta }\left(x,t\right) & =(\Phi_{\delta t}*\exp\left(-f/\delta \right))\left(x\right) \end{array}$$

[12b] $$\begin{array}{cc} & =E_{y∼N(x,\delta t)}\left[\exp\left(-f(y)/\delta \right)\right], \end{array}$$

where *y* ∼ 𝒩(*x*, *δt*) denotes that *y* ∈ ℝ^*n*^ is sampled from a normal distribution with mean *x* and SD $\sqrt{\delta t}$. In practice, finitely many samples *y*^*i*^ ∼ 𝒩(*x*, *δt*) are used to estimate [**12b**]. This can greatly reduce sampling complexity (17, 18). Differentiating *v*^*δ*^ with respect to *x* reveals that

[13] $$\begin{array}{c} \nabla v^{\delta }\left(x,t\right)=-\frac{1}{\delta t}\cdot E_{y∼N(x,\delta t)}\left[\left(x-y\right)\exp\left(-f(y)/\delta \right)\right]. \end{array}$$

Plugging [12b] and [**13**] into [**11**] enables ∇*u*^*δ*^ to be written as

[14] $$\begin{array}{c} \nabla u^{\delta }\left(x,t\right)=\frac{1}{t}\cdot \left(x-\frac{E_{y∼N(x,\delta t)}\left[y\cdot \exp\left(-f(y)/\delta \right)\right]}{E_{y∼N(x,\delta t)}\left[\exp\left(-f(y)/\delta \right)\right]}\right). \end{array}$$

The above relation was used in ref. 19. Here, we take a further step, combining [**4**] and [**14**] to get an HJ-based estimate

[15a] $$\begin{array}{cc} {\mathrm{prox}}_{\mathrm{tf}}\left(x\right) & =x-t\nabla u(x,t) \end{array}$$

[15b] $$\begin{array}{cc} & \approx x-t\nabla u^{\delta }\left(x,t\right) \end{array}$$

[15c] $$\begin{array}{cc} & =\frac{E_{y∼N(x,\delta t)}\left[y\cdot \exp\left(-f(y)/\delta \right)\right]}{E_{y∼N(x,\delta t)}\left[\exp\left(-f(y)/\delta \right)\right]}. \end{array}$$

**Fig. 2.**
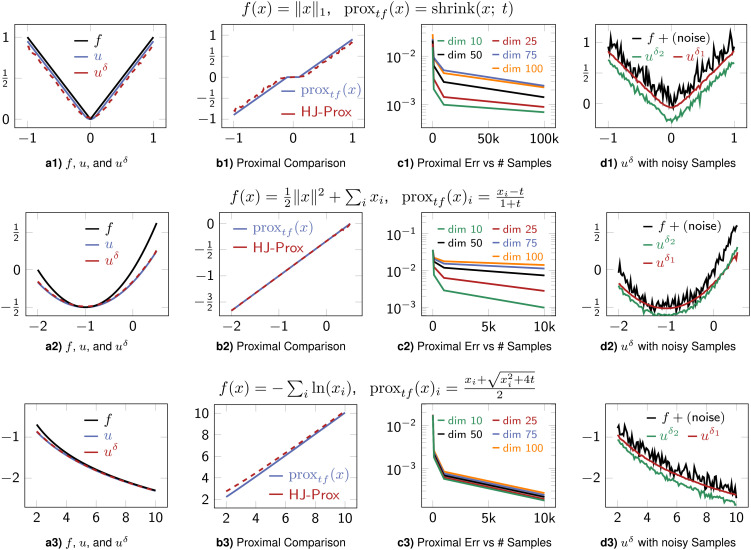
(*a1, a2, a3*): Plots for function *f*, exact Moreau envelope *u*, and HJ-based Moreau envelope *u*^*δ*^. (*b1, b2, b3*): Plots for true proximal and approximate HJ-based proximal operators. (*c1, c2, c3*): Proximal approximations across different dimensions and samples. (*d1, d2, d3*): HJ-based Moreau envelopes *u*^*δ*^ obtained from noisy function samples. Here, we use *δ* = *δ*_1_ = 10^−1^ and *δ*_2_ = 10^−2^. As expected, higher *δ* values have a stronger smoothing property. The HJ-proximals are good approximations of the true proximal operators (seen through the Moreau envelopes) and can be applied even when only (potentially noisy) samples are available. For the noisy case, we obtain a *C*^∞^ approximation of the underlying function *f*. For these experiments, we use *t* = 0.1, 0.5, and 2.0 for rows 1, 2, and 3, respectively.

As shown below, Monte Carlo sampling enables efficient approximation of proximals in high dimensions (e.g., Fig. 2). Moreover, [**15c**] estimates proximals using only function values, making it apt for zeroth-order optimization.

## Numerical Considerations

A possible numerical challenge in our formulation is to address numerical instabilities arising from the exponential term underflowing or overflowing with limited numerical precision due to either *δ* being small or *f*(*y*) being large. Indeed, this makes the naive implementation shown in Algorithm 1numerically unstable. However, this may be remedied as the proximal formula may equivalently be rescaled via

[16a] $$\begin{array}{cc} {\mathrm{prox}}_{\mathrm{tf}}\left(x\right) & ={\mathrm{prox}}_{\frac{t}{\alpha }\alpha f}\left(x\right) \end{array}$$

[16b] $$\begin{array}{cc} & \approx \frac{E_{y∼N(x,\delta t/\alpha )}\left[y\cdot \exp\left(-\alpha f(y)/\delta \right)\right]}{E_{y∼N(x,\delta t/\alpha )}\left[\exp\left(-\alpha f(y)/\delta \right)\right]}, \end{array}$$

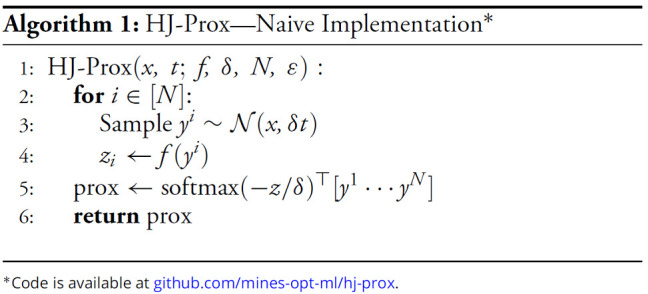

where *t* is replaced by *t*/*α* and *f* by *αf* in [15c]. In this case, if *f*/*δ* becomes too large with respect to numerical precision limitations, it may be scaled down with a corresponding *α*. We can check whether we obtain an underflow with exp(*αf*(*y*)/*δ*) and rescale *α* using a linesearch-like approach (e.g., *SI Appendix* where we add a single conditional statement to recursively halve *α* until exp(*αf*(*y*)/*δ*)> *ε* for a tolerance *ε*). Small *α* makes the variance large, and more samples may be required to accurately estimate the expectations, i.e., a trade-off may be observed between numerical stability and accuracy of estimations. Another possible mitigation is to adaptively rescale *f* based on the number of recursive steps taken in HJ-Prox. Note that a large *δ* can smooth approximations and mitigate the stochastic characteristics of HJ-Prox.

## Convergence Analysis

The arguments above give intuition for a proximal approximation. However, having now the formula [**15c**], we may formalize its utility without reference to differential equations. Below we define a standard class of functions used in optimization.

*Definition 1* (Weakly Convex): For *ρ* > 0, a function *f*: ℝ^*n*^ → ℝ is *ρ*-weakly convex if $f\left(x\right)+\frac{\rho }{2}{\left\|x\right\|}^{2}$ is convex^*^.

Our main result shows that HJ-Prox converges to the proximal.

Theorem 1 (*Proximal Approximation*). *If *f*: ℝ^*n*^ → ℝ is *ρ*-weakly convex, for some *ρ* > 0, and either *L*-Lipschitz or is differentiable with *L*-Lipschitz gradient, then, for all *x* ∈ ℝ^*n*^ and *t* ∈ (0, 1/*ρ*), the proximal prox_*tf*_(*x*) is unique and*

[17] $$\begin{array}{c} \lim_{\delta \to 0^{+}}\frac{E_{y∼N(x,\delta t)}\left[y\cdot \exp\left(-f(y)/\delta \right)\right]}{E_{y∼N(x,\delta t)}\left[\exp\left(-f(y)/\delta \right)\right]}={\mathrm{prox}}_{\mathrm{tf}}\left(x\right). \end{array}$$

A proof of Theorem 1 is in (*SI Appendix*), and we note that HJ-Prox may fail when *f* is discontinuous.

*Remark 1* (Smoothing Property): In practice, we must pick positive *δ*. Thankfully, increasing *δ* comes with the benefit of smoothing estimates (due to the Laplacian in the viscous HJ equation), as shown in Figs. 1 and 2 (*Right* column).

## Related Works

Our proposal closely relates to zeroth-order optimization algorithms, which do not require gradients. In fact, HJ-Prox does not require differentiability of *f*. Related methods include random gradients (21–24), sparsity-based methods (25–27), derivative-free quasi-Newton methods (28–30), finite-difference-based methods (31, 32), numerical quadrature-based methods (33, 34), Bayesian methods (29), and comparison methods (35). As proximals closely relate to the gradient of Moreau envelopes, our work relates to methods that minimize Moreau envelopes (or their approximations) (16, 19, 36–40).

The theoretical result in our work is closely related to the study of asymptotics as *δ* → 0 of integrals containing expressions of the form exp(−*f*/*δ*), i.e., Laplace’s method (2). Moreover, the idea of adding artificial diffusion to Burgers’ equation and then applying Cole–Hopf transformation to approximate the gradient of the solution to the HJ equation has been largely developed in ref. 2 in the context of obtaining solutions to conservation laws in 1D. The connections between Hopf–Lax and Cole–Hopf were first introduced in the context of machine learning in ref. 16 and in the context of global optimization in ref. 19.

## Numerical Experiments

Examples herein show that HJ-Prox (Algorithm 1) can

- approximate proximals and smooth noisy samples,
- converge comparably to existing algorithms, and
- solve a new class of zeroth-order optimization problems.

Each item is addressed by a set of experiments. Regarding the last item, HJ-Prox is a tool that enables faithful solution estimation for constrained problems where the objective is accessible only via noisy black box samples.

### Proximal and Moreau Envelope Estimation.

Herein, we compare HJ-Prox to known proximal operators. Fig. 2 shows HJ-Prox for three functions (absolute value, quadratic, and log barrier) whose proximals are known. In the leftmost column (a), we show the Moreau envelope *u*(*x*, *t*) given by Eq. **2** and an estimate of Moreau envelope using the HJ-Prox *u*^*δ*^(*x*, *t*). Given the close connection between proximals and Moreau envelopes, we believe that this visual is a natural and intuitive way to gauge whether the proximal operator is accurate. Column (b) juxtaposes the true proximal and HJ-Prox. Column (c) shows the accuracy of HJ-Prox across different dimensions and numbers of samples. In the rightmost column (d), we estimate Moreau envelopes using HJ-Prox using noisy function values. The resulting envelopes are smooth since *u*^*δ*^ is a smooth (i.e., *C*^∞^(ℝ^*n*^)) approximation of *u*. Thus, HJ-Prox can be used to obtain smooth estimates from noisy observations.

**Fig. 3.**
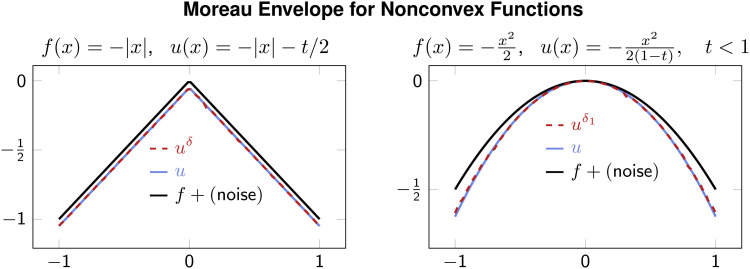
HJ-based Moreau envelope for nonconvex functions with *t* = 0.1 and *t* = 0.2 in the *Left* and *Right* figures, respectively.

**Fig. 4.**
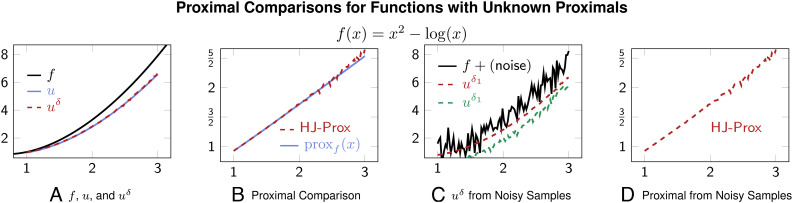
(*A*): Plots for function *f*, exact Moreau envelope *u*, and HJ-based Moreau envelope *u*^*δ*^. (*B*): Plots for true proximal and approximate HJ-based proximal operators. (*C*): HJ-based Moreau envelopes *u*^*δ*^ obtained from noisy function samples. (*D*): HJ-based proximal computed using noisy function samples. Since there is no analytic proximal formula, we obtain the “true” proximal by solving the optimization Eq. **1**) using gradient descent. The HJ-based proximal is a good approximation of the true proximal operators and can be applied even when only (potentially noisy) samples are available. As in the analytic case, we obtain a *C*^∞^ approximation of the underlying function *f* in the noisy case. Here, *δ* = 0.1 for the noiseless case, and *δ*_1_ = 0.5 and *δ*_2_ = 0.1 for the noisy case.

Fig. 3 shows Moreau envelopes for nonconvex functions *f*. As in the other example, here, HJ-based Moreau envelope estimates also accurately approximate Moreau envelopes. Note that these proximals may be well-defined only for small time *t* (as the proximal operator objective in Eq. **1** is strongly convex for small *t*). Lastly, we apply HJ-Prox with a function that has no analytic formula for its proximal or Moreau envelope in Fig. 4. In this experiment, we obtain a “true” Moreau envelope and proximal operator by solving the minimization problem [**1**] iteratively via gradient descent. Faithful recovery is shown in Fig. 4 *A* and *B* and smoothing in Fig. 4*C*.

### Optimization with Proximable Function.

This experiment juxtaposes HJ-prox and an analytic proximal formula in an optimization algorithm. Consider the Lasso problem (41, 42)

[18] $$\underset{x\in {\mathbb{R}}^{1000}}{\min}\begin{array}{c} \frac{1}{2}\|Ax-b{\|}_{2}^{2}+\|x{\|}_{1}, \end{array}$$

where entries of *A* ∈ ℝ^500 × 1000^ and *b* ∈ ℝ^500^ are i.i.d. Gaussian samples. The iterative soft thresholding algorithm (ISTA) (43) defines a sequence of solution estimates {*x*^*k*^} for all *k* ∈ ℕ via

[19] $$\begin{array}{c} x^{k+1}=\;\text{shrink}\left(x^{k}-\beta A^{⊤}\left(Ax^{k}-b\right);\;\beta \right), \end{array}$$

where the shrink operator is defined element-wise by

[20] $$\begin{array}{c} \;\text{shrink}(x;\;t)≜\;\text{sign}(x)\max(0,\;|x|-t). \end{array}$$

**Fig. 5.**
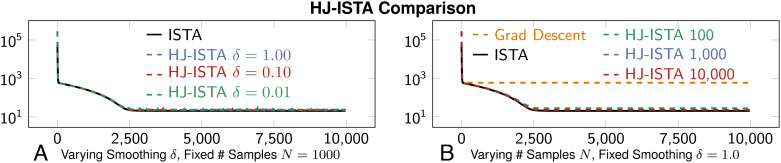
Convergence plots showing function value for solution estimates {*x*^*k*^} when solving the LASSO problem [**18**] with ISTA, juxtaposing use of an analytic proximal formula, gradient descent (i.e., ignoring the proximal), and the approximate HJ-prox (Algorithm 1). Plots with HJ-prox show averaged results from 30 trials with distinct random seeds. To ensure that the proximal is playing a role in the optimization process, we also show a function value history of gradient descent applied to the unregularized least squares problem in Eq. **18** (i.e., with no ℓ_1_ norm term). (*A*) shows performance when varying then smoothing parameter and (*B*) shows performance when varying the number of samples.

Fig. 5 compares the convergence of ISTA using the shrink operator in Eq. **20** and HJ-Prox estimates of the shrink. To ensure convergence, we choose *β* = 1/∥*A*^⊤^*A*∥_2_. Our experiments show that HJ-based ISTA can solve Lasso, up to an error tolerance.

### Optimization with Noisy Objective Oracles.

Consider a constrained minimization problem where objective values *f* can be accessed only via a noisy oracle^†^ 𝒪. Our task is to solve

[21] $$\underset{x\in {\mathbb{R}}^{1000}}{\min}\begin{array}{c} E[O(x)]\;\;\text{s}\text{.t}\text{.}\;Ax=b, \end{array}$$

where *A* and *b* are as in the prior experiment, and the expectation 𝔼 is over oracle noise. To model “difficult” settings (e.g., when a singular value decomposition of *A* is unavailable), we do not use any projections onto the feasible set. Since the solver has no knowledge of the structure of 𝒪, we emphasize schemes for solving [**21**] must use zeroth-order optimization schemes (29). Here, each oracle call returns

[22] $$\begin{array}{c} O\left(x\right)=\left(1+\varepsilon \right)\cdot {\left\|Wx\right\|}_{1},\;\;\text{where}\;\varepsilon ∼N\left(0,\sigma^{2}\right), \end{array}$$

with a new noise sample *ε* ∈ ℝ used in each oracle evaluation, *σ* = 0.005, and *W* ∈ ℝ^1000 × 1000^ a fixed Gaussian matrix. In words, the noise has magnitude 0.5% of ∥*Wx*∥_1_. Although the oracle structure is shown by Eq. **22**, our task is to solve [**21**] without such knowledge. We do this with the linearized method of multipliers (e.g., section 3.5 in ref. 9). Specifically, for each index *k* ∈ ℕ, the update formulas for the solution estimates {*x*^*k*^} and corresponding dual variables {*u*^*k*^} are

[23a] $$\begin{array}{cc} x^{k+1} & ={\mathrm{prox}}_{tO}\left(x^{k}-tA^{⊤}\left(u^{k}+\lambda \left(Ax^{k}-b\right)\right)\right) \end{array}$$

[23b] $$\begin{array}{cc} u^{k+1} & =u^{k}+\lambda \left(Ax^{k+1}-b\right), \end{array}$$

with step sizes *t* = 1/∥*A*^⊤^*A*∥_2_ and *λ* = 1/2. Without noise *ε*, convergence occurs if *tλ*∥*A*^⊤^*A*∥_2_ < 1 (9), justifying our choices for *t* and *λ*. The proximal prox_*t*𝒪_ is estimated by HJ-prox.

**Fig. 6.**
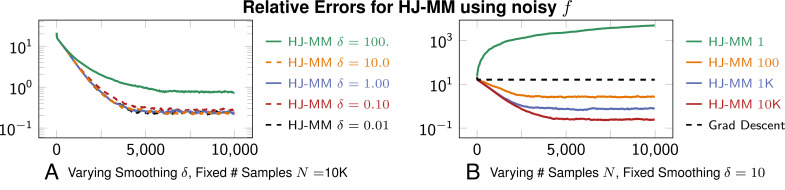
Convergence plots showing relative errors for solution estimates {*x*^*k*^} when solving the minimization problem [**21**] with the linearized method of multipliers and HJ-prox (Algorithm 1). Each plot shows averaged results from 30 trials with distinct random seeds. Due to the noise, we observe in (*A*) that a larger *δ* = 10 leads to a better approximation, but if it is too large (*δ* = 100), it leads to oversmoothing and reduces accuracy. We find *δ* = 10 to be most optimal, and (*B*) shows that more samples lead to more accurate approximations (as expected). To ensure that the proximal is playing a role in the optimization process, we also show the relative error when gradient decent is applied to the constraint residual in Eq. **21** (i.e., we only minimize constraint residual). Indeed, gradient descent performs poorly by comparison. Finally, we note that one recursive iteration (thus doubling the samples) occurs in the first few hundred iterations of HJ-MM. See the algorithm in *SI Appendix* for this recursive scheme.

We separately solve the optimization problem using full knowledge of the objective ∥*Wx*∥_1_ without noise; doing this enables us to plot the relative error of the sequence {*x*^*k*^} in Fig. 6. All the plots show that {*x*^*k*^} converges to the optimal *x*^⋆^, up to an error threshold, regardless of the choice of *δ* and number of samples *N*. Note that Fig. 6*A* shows “small” values of *δ* give comparable accuracy, but that oversmoothing with “large” *δ* = 100 degrades performance of the algorithm. These plots also illustrate that the HJ-prox formula is efficient with respect to calls to the oracle 𝒪. In particular, note the plots in Fig. 6*B* that decrease relative error use at each iteration, respectively use 0.1, 1, and 10 oracle calls per dimension of the problem! We hypothesize that the smoothing effect of the viscous *u*^*δ*^ and averaging effect of Monte Carlo sampling contribute to the observed convergence. In this experiment, HJ-prox converges to within an error tolerance, is efficient with respect to oracle calls, and smooths Gaussian noise.

## Conclusion

We propose an algorithm—HJ-prox—for efficiently approximating proximal operators. This is derived from approximating Moreau envelopes via viscosity solutions to Hamilton–Jacobi (HJ) equations, as given via the Hopf–Lax formula. Upon rewriting this approximation in terms of expectations, we use Monte Carlo sampling to avoid discretizing the integrals, thereby mitigating the curse of dimensionality. Our numerical examples show that HJ-Prox is effective for a collection of functions, both with and without known proximal formulas. Moreover, HJ-prox can be effectively used in constrained optimization problems even when only noisy objective values are available.

## Supplementary Material

Appendix 01 (PDF)

## Data Availability

Code has been deposited in https://github.com/mines-opt-ml/hj-prox (44).

## Footnotes

^*^Weakly convex functions are also referred to as semiconvex functions (20).

^†^Here, 𝒪 is a noisy function, not to be confused with “Big O” often used to describe limit behaviors.
