# Supplementary material for: A Hamilton–Jacobi-based proximal operator
Source: Proc Natl Acad Sci U S A. 2023 Mar 29;120(14):e2220469120. doi: 10.1073/pnas.2220469120 (PMC10083605; doi:10.1073/pnas.2220469120)
Supplement: Supplementary file 1 — Appendix 01 (PDF) [file pnas.2220469120.sapp.pdf]

Supporting Information: A Hamilton-Jacobi-based Proximal Operator

Stanley Osher[a,1], **Howard Heaton**[b,1], **and Samy Wu Fung**[c,1,2]

[a]Dept. of Mathematics, University of California, Los Angeles; [b]Typal Research, Typal LLC; [c]Dept. of Applied Mathematics and Statistics, Colorado School of Mines

HJ-Prox Implementation

Below we provide a more numerically stable HJ-Prox implementation that avoids underflow.

Algorithm 1 HJ-Prox – IncludesUnderflow Check

```
HJ-Prox(x, t; f, δ, N, α, ε) :
  for i ∈ [N]:
    Sample y^i ∼ N(x, δt/α)
    z_i ← f(y^i)
    if exp(−α z_i/δ) ≤ ε:
      return HJ-Prox(x, t; f, δ, N, α/2, ε)
  prox ← softmax(−αz/δ)^⊤ [y^1 · · · y^N]
  return prox
```

Proofs

We fix the point $x \in \mathbb{R}^n$ for the following calculations. For concise expression below, for $t > 0$ and $\delta > 0$, define

$$\phi_t(z) \triangleq f(z) + \frac{1}{2t}\|z - x\|^2, \qquad [1]$$

$\phi_t^\star \triangleq \inf\{\phi_t(y) : y \in \mathbb{R}^n\}$, $\xi^\star \triangleq \operatorname{prox}_{tf}(x) = \operatorname{argmin} \phi_t$ (*n.b.* existence and uniqueness of the minimizer $\xi^\star$ are shown below), and

$$\sigma_\delta(z) \triangleq \frac{\exp\left(-\phi_t(z)/\delta\right)}{\|\exp(-\phi_t/\delta)\|_{L^1(\mathbb{R}^n)}} = \frac{e^{-\frac{\phi_t(z)}{\delta}}}{\|e^{-\frac{\phi_t}{\delta}}\|_{L^1(\mathbb{R}^n)}}. \qquad [2]$$

Lemma 1. *If the conditions of Theorem 1 hold, then*

$$\int_{\mathbb{R}^n} \sigma_\delta(y)\ \mathrm{d}y = 1, \quad \sigma_\delta(y) \geq 0, \quad \text{for all } y \in \mathbb{R}^n, \qquad [3]$$

and, for all $r \in (0,1)$ and polynomials p of positive degree,

$$\lim_{\delta\to 0^+} \int_{\mathbb{R}^n - \mathcal{B}(\xi^\star, r)} \sigma_\delta(y) p(\|y - \operatorname{prox}_{tf}(x)\|)\ dy = 0, \qquad [4]$$

where $\operatorname{prox}_{tf}(x)$ is the unique minimizer of ϕ_t.

Proof. Since integration is linear and the limit of a sum is the sum of the limits, it suffices to verify [4] for any $p(x) = x^k$ with $k \geq 1$. First we show σ_δ satisfies properties to be a probability density (Step 1). We also show various L^p norm limits hold for the denominator (Step 2) and numerator(Step 3) of the integrand in [4]. Combining these limits gives [4] (Step 4).

Step 1 The numerator and denominator in the definition [2] for σ_δ are nonnegative, making $\sigma_\delta \geq 0$ everywhere. By the choice of t, ϕ_t is $\theta \triangleq 1/t - \rho$ strongly convex, and so it admits a unique minimizer $\xi^\star = \operatorname{prox}_{tf}(x)$ and satisfies

$$\phi_t(y) \geq \phi_t^\star + \langle 0, y - \xi^\star\rangle + \frac{\theta}{2}\|y - \xi^\star\|^2, \text{ for all } y \in \mathbb{R}^n. \qquad [5]$$

Consequently,

$$0 < e^{-\frac{\phi_t(y)}{\delta}} \leq e^{-\frac{\phi_t^\star + \frac{\theta}{2}\|y-\xi^\star\|^2}{\delta}}, \text{ for all } y \in \mathbb{R}^n. \qquad [6]$$

Since the upper bound above is an exponential that decays quadratically (*i.e.* a Gaussian), the middle term in [6] is integrable over $\mathbb{R}^n$, and so the denominator in the definition of σ_δ is positive and finite. Thus, [3] readily follows as the integral of the numerator of σ_δ equals the denominator of σ_δ.

Step 2 A classic result in analysis (*e.g.* see (1, Exercise 3.4)) states L^p norms converge to the L^∞ norm as $p \to \infty$, and so

$$\lim_{\delta\to 0^+} \left\|e^{-\phi_t}\right\|_{L^{\frac{1}{\delta}}(\mathbb{R}^n)} = \|e^{-\phi_t}\|_{L^\infty(\mathbb{R}^n)} = e^{-\phi_t^\star}, \qquad [7]$$

where, for all $\delta > 0$, the $L^{\frac{1}{\delta}}$ norm is finite by Step 1 and the final equality holds since $\phi_t^\star$ is the infimum of ϕ_t.

Step 3 Integrating the numerator of [4] (*i.e.* not including division by the L^1 norm in the definition of σ_δ) for $p(x) = x^k$ gives

$$\int_{\mathbb{R}^n - \mathcal{B}(\xi^\star, r)} e^{-\frac{\phi_t(y)}{\delta}} \|y - \xi^\star\|^k\ \mathrm{d}y \qquad [8a]$$

$$\leq \int_r^\infty e^{-\frac{\phi_t^\star + \frac{\theta\tau^2}{2}}{\delta}} \tau^k \cdot n|\mathcal{B}(\xi^\star, 1)|\tau^{n-1}\ \mathrm{d}\tau \qquad [8b]$$

$$= n|\mathcal{B}(\xi^\star, 1)| \cdot \int_r^\infty e^{-\frac{\phi_t^\star + \frac{\theta\tau^2}{2} - (n+k-1)\ln(\tau^\delta)}{\delta}}\ \mathrm{d}\tau, \qquad [8c]$$

where the first inequality holds by a change of variables to polar coordinates and using the strong convexity of ϕ_t in [5], and the final line holds by properties of logarithms.

Now define

$$\varepsilon \triangleq \frac{\theta}{4(n+k-1)} > 0, \qquad [9]$$

where the denominator is positive since $n \geq 1$ and p has positive degree (*i.e.* $k \geq 1$). For all $0 < \delta < \varepsilon$, observe

$$\tau > 1 \quad \Longrightarrow \quad \tau^\delta < \tau^\varepsilon \quad \text{and} \quad \tau \leq 1 \quad \Longrightarrow \quad \tau^\delta \leq 1^\varepsilon, \quad [10]$$

i.e.

$$\tau^\delta \leq \max(\tau, 1)^\varepsilon, \quad \text{for all } \delta \in (0, \varepsilon). \quad [11]$$

Whence, rewriting [8], we deduce, for all $\delta \in (0, \varepsilon)$,

$$\frac{1}{n|\mathcal{B}(\xi^\star, 1)|} \cdot \int_{\mathbb{R}^n - \mathcal{B}(\xi^\star, r)} e^{-\frac{\phi_t(y)}{\delta}} \|y - \xi^\star\|^k \ \mathrm{d}y \quad [12a]$$

$$\leq \int_r^\infty e^{-\frac{\phi_t^\star + \frac{\theta\tau^2}{2} - \varepsilon(n+k-1)\ln(\max(\tau,1))}{\delta}} \ \mathrm{d}\tau. \quad [12b]$$

Let $q(\tau)$ be the numerator inside the exponential in the integrand of [12b]. Taking the limit yields

$$\lim_{\delta \to 0^+} \|e^{-q}\|_{L^{\frac{1}{\delta}}([r,\infty))} = \left\|e^{-q}\right\|_{L^\infty([r,\infty))}. \quad [13]$$

Let $\tau^\star$ be the minimizer of q over $[r, \infty)$. If $\tau^\star > 1$, then the first order necessary condition and [9] together imply

$$0 = \theta\tau^\star - \frac{\varepsilon(n+k-1)}{\tau^\star} \quad [14]$$

and so

$$\tau^\star = \sqrt{\frac{\varepsilon(n+k-1)}{\theta}} = \frac{1}{2}, \quad [15]$$

a contradiction (*n.b.* the second equality holds by choice of ε in [9]). Consequently, $\tau^\star \leq 1$. Since q is quadratic in τ and strictly increasing on $[r, 1)$, we deduce $\tau^\star = r$. Thus,

$$\|e^{-q}\|_{L^\infty([r,\infty))} = e^{-\phi_t^\star - \frac{\theta r^2}{2}}. \quad [16]$$

Furthermore, note

$$\lim_{\delta \to 0^+} \left[n|\mathcal{B}(\xi^\star, 1)|\right]^\delta = 1. \quad [17]$$

Together [12], [16], and [17] imply

$$\lim_{\delta \to 0^+} \left[\int_{\mathbb{R}^n - \mathcal{B}(\xi^\star, r)} e^{-\frac{\phi_t(y)}{\delta}} \|y - \xi^\star\|^k \ \mathrm{d}y\right]^\delta \leq e^{-\phi_t^\star - \frac{\theta r^2}{2}}. \quad [18]$$

Step 4 Define

$$\gamma \triangleq \frac{e^{-\phi_t^\star - \frac{\theta r^2}{2}}}{e^{-\phi_t^\star}} \in (0, 1). \quad [19]$$

By [7] and [18] and the definition of σ_δ,

$$\lim_{\delta \to 0^+} \left[\int_{\mathbb{R}^n - \mathcal{B}(\xi^\star, r)} \sigma_\delta(y) \|y - \xi^\star\|^k \ \mathrm{d}y\right]^\delta \leq \gamma < 1. \quad [20]$$

Consequently, there is $\bar{\delta} > 0$ such that, for all $\delta \in (0, \bar{\delta}]$,

$$\left[\int_{\mathbb{R}^n - \mathcal{B}(\xi^\star, r)} \sigma_\delta(y) \|y - \xi^\star\|^k \ \mathrm{d}y\right]^\delta \leq \frac{\gamma + 1}{2}, \quad [21]$$

where we note $(\gamma + 1)/2 \in (\gamma, 1)$, and so

$$\lim_{\delta \to 0^+} \int_{\mathcal{S}} \sigma_\delta(y) \|y - \xi^\star\|^k \ \mathrm{d}y \leq \lim_{\delta \to 0^+} \left(\frac{\gamma+1}{2}\right)^{1/\delta} \quad [22a]$$

$$= 0, \quad [22b]$$

as desired. □

Lemma 2. *If the conditions of Theorem 1 hold, then there are constants $a > 0$ and $b \geq 0$ such that ϕ_t has an upper bound of the form, for all $y \in \mathbb{R}^n$,*

$$\phi_t(y) \leq a\|y - \text{prox}_{tf}(x)\|^2 + b\|y - \text{prox}_{tf}(x)\| + \phi_t^\star, \quad [23]$$

where $\text{prox}_{tf}(x)$ is the unique minimizer of ϕ_t.

Proof. For notational compactness, set $\xi^\star = \text{prox}_{tf}(x)$, and note $\xi^\star$ exists and is unique by Lemma 1. We first verify the statement for L-Lipschitz f (Step 1) and then for when the gradient of f is L-Lipschitz (Step 2).

Step 1 Suppose f is L-Lipschitz for some $L > 0$, *i.e.*

$$\|f(y) - f(z)\| \leq L\|y - z\|, \quad \text{for all } y, z \in \mathbb{R}^n. \quad [24]$$

Next note, for all $y \in \mathbb{R}^n$,

$$\|y - x\|^2 - \|\xi^\star - x\|^2 \quad [25a]$$

$$= \|y\|^2 - \|\xi^\star\|^2 - 2\left\langle y - \xi^\star, x\right\rangle \quad [25b]$$

$$\leq (\|\xi^\star\| + \|y - \xi^\star\|)^2 - \|\xi^\star\|^2 + 2\|y - \xi^\star\|\|x\| \quad [25c]$$

$$= \|y - \xi^\star\|^2 + 2\|y - \xi^\star\|(\|\xi^\star\| + \|x\|). \quad [25d]$$

Consequently, [24] and [25] together imply, for all $y \in \mathbb{R}^n$,

$$\phi_t(y) - \phi_t^\star \quad [26a]$$

$$= f(y) - f(\xi^\star) + \frac{1}{2t}\left[\|y - x\|^2 - \|\xi^\star - x\|^2\right] \quad [26b]$$

$$\leq L\|y - \xi^\star\| \quad [26c]$$

$$+ \frac{1}{2t}\left[\|y - \xi^\star\|^2 + 2\|y - \xi^\star\|(\|\xi^\star\| + \|x\|)\right]. \quad [26d]$$

Thus, the upper bound in [23] holds with

$$a = \frac{1}{2t} \quad \text{and} \quad b = \frac{\|\xi^\star\| + \|x\|}{t} + L. \quad [27]$$

Step 2 Consider when f has an L-Lipschitz gradient for some $L > 0$. By (2, Lemma 5.7), for all $y \in \mathbb{R}^n$,

$$f(y) \leq f(\xi^\star) + \langle \nabla f(\xi^\star), y - \xi^\star \rangle + \frac{L}{2}\|y - \xi^\star\|^2 \quad [28a]$$

$$\leq f(\xi^\star) + L\|y - \xi^\star\| + \frac{L}{2}\|y - \xi^\star\|^2. \quad [28b]$$

Rearranging and again using [25] implies

$$\phi_t(y) - \phi_t^\star \quad [29a]$$

$$\leq L\|y - \xi^\star\| + \frac{L}{2}\|y - \xi^\star\|^2 \quad [29b]$$

$$+ \frac{1}{2t}\left[\|y - \xi^\star\|^2 + 2\|y - \xi^\star\|(\|\xi^\star\| + \|x\|)\right] \quad [29c]$$

Thus, the upper bound in [23] holds with

$$a = \frac{1}{2}\left(\frac{1}{t} + L\right) \quad \text{and} \quad b = L + \frac{\|\xi^\star\| + \|x\|}{t}. \quad [30]$$

This completes both cases of the proof. □

Below we restate and prove the main theorem, which is an extension of a lemma in Section 4.5.2 of (3).

Theorem 1 (Proximal Approximation). *If $f\colon \mathbb{R}^n \to \mathbb{R}$ is ρ-weakly convex, for some $\rho > 0$, and either L-Lipschitz or is differentiable with L-Lipschitz gradient, then, for all $x \in \mathbb{R}^n$ and $t \in (0, 1/\rho)$, the proximal $\mathrm{prox}_{tf}(x)$ is unique and*

$$\lim_{\delta\to 0^+} \frac{\mathbb{E}_{y\sim\mathcal{N}(x,\delta t)}\left[y\cdot \exp\left(-f(y)/\delta\right)\right]}{\mathbb{E}_{y\sim\mathcal{N}(x,\delta t)}\left[\exp\left(-f(y)/\delta\right)\right]} = \mathrm{prox}_{tf}(x). \quad [31]$$

Proof. Let $x \in \mathbb{R}^n$ and $t > 0$ be given. For notational compactness, denote the HJ-prox formula by

$$\xi^\delta \triangleq \frac{\mathbb{E}_{y\sim\mathcal{N}(x,\delta t)}\left[y\cdot \exp\left(-f(y)/\delta\right)\right]}{\mathbb{E}_{y\sim\mathcal{N}(x,\delta t)}\left[\exp\left(-f(y)/\delta\right)\right]}, \quad \text{for all } \delta > 0, \quad [32]$$

denote the proximal by $\xi^\star \triangleq \mathrm{prox}_{tf}(x)$, and note $\phi_t^\star = \phi_t(\xi^\star)$. As argued in Lemma 1, $\xi^\star$ is well-defined. We first bound $\phi_t - \phi_t^\star$ using Jensen's inequality (Step 1). Second, we show $\phi_t(\xi^\delta) \to \phi_t(\xi^\star)$ (Step 2). The strong convexity of ϕ_t enables us to establish the desired limit (Step 3).

Step 1 Note ξ^δ can be rewritten via

$$\xi^\delta = \left[\int_{\mathbb{R}^n} e^{-\frac{\phi_t(y)}{\delta}}\, \mathrm{d}y\right]^{-1} \int_{\mathbb{R}^n} y\cdot e^{-\frac{\phi_t(y)}{\delta}}\ \mathrm{d}y. \quad [33]$$

Using σ_δ, the estimate can be more concisely written via

$$\xi^\delta = \int_{\mathbb{R}^n} \sigma_\delta(y) y\ \mathrm{d}y = \mathbb{E}_{y\sim\mathbb{P}_{\sigma_\delta}}\left[y\right], \quad [34]$$

where the expectation holds by utilizing the fact [3] shows σ_δ defines a probability density. Thus, Jensen's inequality may be applied to deduce

$$\phi_t^\star \le \phi_t(\xi^\delta) = \phi_t\left(\mathbb{E}_{y\sim\sigma_\delta}[y]\right) \le \mathbb{E}_{y\sim\sigma_\delta}\left[\phi_t(y)\right]. \quad [35]$$

In integral form, we may subtract $\phi_t^\star$ to write

$$0 \le \phi_t(\xi^\delta) - \phi_t^\star \le \int_{\mathbb{R}^n} \sigma_\delta(y)[\phi_t(y) - \phi_t^\star]\ \mathrm{d}y. \quad [36]$$

Step 2 Let $\varepsilon > 0$ be given. To deduce $\phi_t(\xi^\delta) \to \phi_t^\star$, we verify there is $\delta^\star > 0$ such that

$$|\phi_t(\xi^\delta) - \phi_t^\star| \le \varepsilon, \quad \text{for all } \delta \in (0, \delta^\star]. \quad [37]$$

By [36], the relation [37] holds if there is such a $\delta^\star$ that

$$\int_{\mathbb{R}^n} \sigma_\delta(y)[\phi_t(y) - \phi_t^\star]\ \mathrm{d}y \le \varepsilon, \quad \text{for all} \quad \delta \in (0, \delta^\star]. \quad [38]$$

We verify this by splitting the integral into two parts. By Lemma 2, the fact f is either L-Lipschitz or L-smooth implies there is $a > 0$ and $b \ge 0$ such that, for all $y \in \mathbb{R}^n$,

$$\phi_t(y) - \phi_t^\star \le a\|y - \xi^\star\|^2 + b\|y - \xi^\star\|. \quad [39]$$

Fix $r \in (0, 1)$ sufficiently small to ensure

$$r(ar + b) = ar^2 + br \le \frac{\varepsilon}{2}. \quad [40]$$

This implies

$$\phi_t(y) - \phi_t^\star \le a\|y - \xi^\star\|^2 + b\|y - \xi^\star\| \quad [41a]$$
$$\le \frac{\varepsilon}{2}, \quad \text{for all } y \in \mathcal{B}(\xi^\star, r). \quad [41b]$$

Thus, integrating over the ball $\mathcal{B}(\xi, r)$ reveals

$$A \triangleq \int_{\mathcal{B}(\xi^\star, r)} \sigma_\delta(y)[\phi_t(y) - \phi_t^\star]\ \mathrm{d}y \quad [42a]$$
$$\le \int_{\mathcal{B}(\xi, r)} \sigma_\delta(y)\cdot\frac{\varepsilon}{2}\ \mathrm{d}y \quad [42b]$$
$$\le \frac{\varepsilon}{2}\cdot\int_{\mathbb{R}^n} \sigma_\delta(y)\ \mathrm{d}y \quad [42c]$$
$$= \frac{\varepsilon}{2}, \quad [42d]$$

where the second inequality follows from [3]. Next we integrate over the rest of $\mathbb{R}^n$. Define

$$B_\delta \triangleq \int_{\mathbb{R}^n - \mathcal{B}(\xi^\star, r)} \sigma_\delta(y)[\phi_t(y) - \phi_t^\star]\ \mathrm{d}y \quad [43a]$$
$$\le \int_{\mathbb{R}^n - \mathcal{B}(\xi^\star, r)} \sigma_\delta(y)\cdot p(\|y - \xi^\star\|)\ \mathrm{d}y. \quad [43b]$$

By Lemma 1, there is $\omega > 0$ such that

$$B_\delta \le \frac{\varepsilon}{2}, \quad \text{for all } \delta \in (0, \omega]. \quad [44]$$

Consequently, [42] and [44] together imply

$$\int_{\mathbb{R}^n} \sigma_\delta(y)[\phi_t(y) - \phi_t^\star]\ \mathrm{d}y = A + B_\delta \quad [45a]$$
$$\le \frac{\varepsilon}{2} + \frac{\varepsilon}{2} \quad [45b]$$
$$\le \varepsilon, \quad \text{for all } \delta \in (0, \omega]. \quad [45c]$$

Hence [38] holds, taking $\delta^\star = \omega$. That is, we obtain the convergence $\phi_t(\xi^\delta) \to \phi_t^\star$ as $\delta \to 0^+$.

Step 3 Let $\overline{\varepsilon} > 0$. It suffices to show there is $\overline{\delta} > 0$ such that

$$\|\xi^\delta - \xi^\star\| \le \overline{\varepsilon}, \quad \text{for all } \delta \in (0, \overline{\delta}]. \quad [46]$$

Define

$$\mathcal{S} \triangleq \{z : \|z - \xi^\star\| \ge \overline{\varepsilon}\} \quad [47]$$

and note, by the strong convexity of ϕ_t (*e.g.* see [5]),

$$\phi_t(z) \ge \phi_t^\star + \frac{\theta\overline{\varepsilon}^2}{2}, \quad \text{for all } z \in \mathcal{S}. \quad [48]$$

By Step 2, there is $\mu > 0$ such that

$$\phi_t(\xi^\delta) \le \phi_t^\star + \frac{\theta\overline{\varepsilon}^2}{4}, \quad \text{for all } \delta \in (0, \mu]. \quad [49]$$

Thus, $\xi^\delta \notin \mathcal{S}$, for all $\delta \in (0, \mu]$, *i.e.* (46) holds, taking $\overline{\delta} = \mu$. This completes the proof. ☐

1. W Rudin, *Real and Complex Analysis.* (McGraw-Hill), (1966).
2. A Beck, *First-order methods in optimization.* (SIAM), (2017).
3. LC Evans, Partial Differential Equations. *Graduate Stud. Math.* **19** (2010).
